# More eyes on the prize: open-source data, software and hardware for advancing plant science through collaboration

**DOI:** 10.1093/aobpla/plad010

**Published:** 2023-03-09

**Authors:** Guy R Y Coleman, William T Salter

**Affiliations:** School of Life and Environmental Sciences, Sydney Institute of Agriculture, The University of Sydney, Brownlow Hill, New South Wales 2570, Australia; School of Life and Environmental Sciences, Sydney Institute of Agriculture, The University of Sydney, Narrabri, New South Wales 2390, Australia

**Keywords:** Computer vision, machine learning, open-source, phenotyping, site-specific weed control, sustainable agriculture

## Abstract

Automating the analysis of plants using image processing would help remove barriers to phenotyping and large-scale precision agricultural technologies, such as site-specific weed control. The combination of accessible hardware and high-performance deep learning (DL) tools for plant analysis is becoming widely recognised as a path forward for both plant science and applied precision agricultural purposes. Yet, a lack of collaboration in image analysis for plant science, despite the open-source origins of much of the technology, is hindering development. Here, we show how tools developed for specific attributes of phenotyping or weed recognition for precision weed control have substantial overlapping data structure, software/hardware requirements and outputs. An open-source approach to these tools facilitates interdisciplinary collaboration, avoiding unnecessary repetition and allowing research groups in both basic and applied sciences to capitalise on advancements and resolve respective bottlenecks. The approach mimics that of machine learning in its nascence. Three areas of collaboration are identified as critical for improving efficiency, (1) standardized, open-source, annotated dataset development with consistent metadata reporting; (2) establishment of accessible and reliable training and testing platforms for DL algorithms; and (3) sharing of all source code used in the research process. The complexity of imaging plants and cost of annotating image datasets means that collaboration from typically distinct fields will be necessary to capitalize on the benefits of DL for both applied and basic science purposes.

## Teaser

Using image processing to automate plant analysis can help overcome barriers in precision agriculture and plant science for improved crop breeding. Despite shared goals and similar methods for image-based plant analysis, there is limited collaboration and sharing of tools, holding back progress. The field of machine learning has demonstrated how open-source and collaborative approaches result in rapid improvement. An open-source approach to tools for plant phenotyping or weed recognition can facilitate collaboration, avoid duplication and improve research reach and efficiency. Three critical areas of collaboration are identified: developing standardized, open-source, labeled datasets; creating accessible and reliable platforms for training and testing deep learning algorithms; and sharing all source code used in research.

## Introduction

The automated detection and extraction of contextual information about individual plants from imaging sensors has the potential to transform plant science research and precision agricultural practices. The value of such techniques stems from reducing labour and the coarseness of spatial and temporal assessments in fields such as crop phenotyping and site-specific weed control (SSWC). In plant phenotyping, this results in more affordable and quantitative plant breeding screening pipelines and improved understanding of crop physiology, development, and anatomy (Singh *et al.*, 2018). In SSWC, the benefits arise from confidently identifying individual weeds and targeting the weed control method to each plant directly (i.e. spot spraying or targeted tillage), instead of indiscriminate application. This results in reduced input costs and new management opportunities (Slaughter *et al.*, 2008; Coleman *et al.*, 2019). For example, the use of non-imaging, reflectance-based weed detection systems for spot spraying in fallow weed control scenarios have reduced herbicide usage by up to 90% (Timmermann *et al.*, 2003). The savings observed in fallow settings have generated substantial interest in the use of weed recognition technologies for more complex in-crop scenarios.

The term ‘phenotype’ was formed by Danish plant scientist Wilhelm Johannsen meaning: ‘*all “types” of organisms, distinguishable by direct inspection or only by finer methods of measuring or description’* (Johannsen, 1911). Over 110 years later, phenotyping is a highly complex process, broadly referring to a suite of assessments conducted to characterise the morphological, physiological, biochemical, ontogenetical and ecological traits of a plant (Walter *et al.*, 2015). In plant sciences, plant phenotyping is widely regarded as a major bottleneck in the development of new crop varieties and for the understanding of plant traits and responses (Furbank and Tester, 2011; Fahlgren *et al.*, 2015; Araus and Kefauver, 2018; Li *et al.*, 2020), whereby the measurement of plant traits constrains the potential advancements made possible by genetic improvements (Sarić *et al.*, 2022). The replacement of existing qualitative and laborious methods not only generates potential for improving rigour and saving costs, but transforming crop domestication and breeding from novel data with increased spatial, spectral, temporal and population-level resolution that unlocks the unrealised potential of genetic variation in traits of interest (Danilevicz *et al.*, 2021; Van Tassel *et al.*, 2022).

A logical extension of Johannsen’s definition is the real-time ‘phenotyping’ of a crop or field for the differentiation, detection and control of weeds. In this case, the ‘*direct inspection*’ is by a sensor and rather than measuring traits for breeding or selection, the output is some form of control. Similarly to phenotyping for plant breeding, the reliable detection of weeds and individual plant organs is identified as the biggest barrier to widespread deployment and reliable operation of in-crop SSWC tools (Lopez-Granados, 2011; Fernández-Quintanilla *et al.*, 2018; Hasan *et al.*, 2021). The shared challenge of reliable plant imaging and analysis (and identified downstream opportunities) is certainly not new, with attempts in both the weed control and plant science fields to achieve reliable plant recognition spanning almost a half-century (Hooper *et al.*, 1976; Guyer *et al.*, 1986; Shearer and Holmes, 1990). Much of the difficulty in relying on image-based plant recognition systems arises in the variability and complexity of environmental conditions in which plants grow and the diversity of plant appearances (Jiang and Li, 2020; Hasan *et al.*, 2021).

The path to unlock the potential offered by image-based plant analysis has profited deeply from advancements made in computer vision. Up until 2012, popular computer vision algorithms were based around the manual extraction of features and ‘non-deep’ machine learning algorithms. This meant ‘experts’ would identify important plant attributes, for example, shape, size and colour for the task at hand (Singh *et al.*, 2018). However, in 2012, a convolutional neural network (CNN) outcompeted other non-CNN algorithms on the ImageNet large-scale visual recognition challenge (ILSVRC), setting in motion a new era of CNN-focused image-analysis (Krizhevsky *et al.*, 2012). The ILSVRC highlights the benefits of benchmarking (as identified for plant science in Danilevicz *et al.*, 2021), where machine learning engineers used a standardised dataset to compare classification performance on approximately 22 000 categories from over 15 million images (Deng *et al.*, 2009). In Krizhevsky’s work, the automation of the feature extraction process brought about through the CNN provided new ways of increasing algorithm robustness to variability, removing the subjectivity of human judgment from the feature extraction process, a dominant feature of prior ILSVRC attempts. Moreover, the large, complex nature of deep learning (DL) architectures mean they are more capable of representing intricacies in data than ‘non-deep’ methods (Bengio *et al.*, 2013). Since their first use for leaf identification in the mid-2010s (Lee *et al.*, 2015), DL algorithms have dramatically risen in popularity, becoming the dominant tool for plant image analysis and weed recognition (Singh *et al.*, 2018; Jiang and Li, 2020; Hasan *et al.*, 2021). An underlying force behind development in DL has been the principles of open-source software (Box 1). This framework includes open access to large image datasets on which to develop and compare new algorithms, open-source software with which to train and test models, and open accessibility of the algorithm architectures themselves on which to build and iterate (Sonnenburg *et al.*, 2007).

**Box 1 Identifying a Need for Collaboration in Machine Learning**

The discipline of machine learning has not always been an example of effective open-source development. In 2007, Sonnenburg *et al*. identified that research efficiency and advancement in the field was limited by a lack of sharing of algorithm implementations and/or source code. The authors identified seven advantages to the discipline of an open-source approach:

1. reproducibility and fair comparison;
2. identifying issues and errors;
3. building on rather than repeating the development of tools and resources;
4. access to scientific tools;
5. combining developments;
6. faster adoption in external disciplines; and
7. collaborative development of standards.

Almost 15 years later, the very fact that this article focused on deep learning is being published in a plant science journal is testament to the wide-reaching impact of the adoption of these ideals by the machine learning community. A likely reason for the popularity of deep learning-based methods in plant sciences is that open-source development has dramatically lowered the barrier to entry for non-computer science users.

Image data used for phenotyping and weed recognition can be collected from a range of sensors including visual, multi- and hyperspectral imaging devices. Digital camera technology is widely available, with sensitivity to light in the 400 to 1000 nm range across broad-spectrum red, green and blue (RGB) bands. Infrared blocking filters are included in most consumer cameras on the red band, to restrict sensitivity to visible light only (Grieder *et al.*, 2015). Multi- and hyperspectral imagers provide targeted sensitivity to specific wavelengths, enabling the use of vegetation indices for abiotic and biotic stress detection (Fahlgren *et al.*, 2015). However, the choice of image data used, either visual, multi or hyperspectral, is generally influenced by the accessibility of the device (i.e., cost, ease of use, appropriateness for field work and availability of established analysis pipelines), the measured traits and the performance advantage of more complex multi- and hyperspectral devices. RGB sensors have gained popularity, driven in part by the success of visual-spectrum image analysis, the accessibility of high-resolution digital imaging sensors, and the need to efficiently capture large image datasets for training DL algorithms. Similarly, there has been a shift towards the use of RGB cameras in weed recognition (Peteinatos *et al.*, 2020). The increasing accessibility of RGB cameras and low cost capture of image data is critical, given a major limitation of DL algorithms is the requirement for large quantities of annotated images (Lu and Young, 2020).

Reviews in both phenotyping and weed recognition draw similar conclusions from the body of research specific to the discipline, yet often without acknowledgement or use of crossover research. The requirement of DL-based CNNs to access substantial quantities of training image data and the development of expensive processing pipelines are often identified as limiting factors in the research (Table 1). For example, conclusions of reviews from Wang *et al.* (2019) on computer vision-based weed recognition and Li *et al.* (2020) on computer vision-based plant phenotyping, were that data availability and robustness in variable conditions were current bottlenecks. Lu and Young (2020) concluded that most weeds-related datasets were small and the task of weed recognition was highly variable, requiring many more images for robust performance. Araus and Kefauver (2018) concur, going further to identify that effective phenotyping is strongly reliant on the entire pipeline of data collection platforms, data throughput, variability management and the integration of the data and decisions with the research program. These identified areas share many similarities with the four principles of SSWC described by Lopez-Granados (2011) as (1) weed detection and monitoring, (2) decision making, (3) execution in the field and (4) evaluation of SSWC impacts (profitability, safety, environmental). Research that explicitly describes the cross-applicability of their findings is rare, though Jiang and Li (2020) touched on the shared bottleneck of interpreting individual plants on a large-scale. Danilevicz *et al.* (2021) provide detail from different disciplines with potential to improve plant phenotyping, recommending that for high throughput plant phenotyping to flourish there should be collaborative efforts for accessible and standardised benchmarking datasets.

**Table 1.** Recommendations developed in recent reviews from phenotyping and weed recognition. Included are the conclusions highlighted by the authors as critical to improving the use of/access to deep learning. Different conclusions are coloured based on fit within the four stages of the deep learning pipeline (Fig. 1) and shaded based on similarity between reviews. The crossover between the two fields with respect to data standards and access is clear, despite the differences and generally lack of inclusion of references from each discipline in the other review. As expected, differences in conclusions appear in the end use of the technology.

	Phenotyping reviews					Weed recognition reviews				
	Singh *et al.* (2018)	Jiang and Li (2020)	Li *et al.* (2020)	Danilevicz *et al.* (2021)	Wang and Su (2022)	Wang *et al.* (2019)	Hasan *et al.* (2021)	Rakhmatulin *et al.* (2021)	Wu *et al.* (2021)	Gerhards *et al.* (2022)
Data (collection, processing and storage)	Creating community-curated accessible datasets Collecting imaging data in relevant field conditions	Improving availability of diverse, annotated datasets	Establishing multi-domain/level/scale plant phenotypic database	Developing public repositories (with metadata Developing benchmarking datasets Developing dataset standards	Improving access to high quality images Improving dataset annotation efficiency Including multi- and hyperspectral data	Improving availability of large and diverse image datasets Reducing laborious nature of annotation Developing benchmarking datasets	Improving availability of large and diverse image datasets Improving management of class imbalance Developing benchmarking datasets	Using open-access benchmarking datasets to compare algorithms	Improving access to datasets used in the study Managing diversity of field conditions	Establishing an open-access image database
Algorithm		Customising deep learning frameworks to improve adoption		Adopting federative learning to avoid data sharing issues	Developing new plant detection architectures			Sharing source code Improving detection of small objects	Developing fast and high performing algorithms	
Deployment					Developing lightweight algorithms for mobile devices	Balancing image processing time with performance	Efficient detection for real-time use	Balancing algorithm complexity with processing capacity		Standardising communication
Output		Developing algorithms for 3D plant phenotyping	Developing trait identification Integrating with bioinformatics							Species-targeted herbicide use Improving in-row mechanical weeding

A root cause of the problem appears in literature search terms often reported in reviews, which restrict the review scope on semantic differences, rather than the qualities and utility of the body of research to the intended review field. In a comprehensive review of literature for DL based weed recognition, Hasan *et al.* (2021) uses Boolean search queries for databases such as Scopus and Google Scholar that generally fit a ‘(*Weed Detection OR Weed management OR Weed Classification) AND (Deep Learning OR Deep Machine Learning OR Deep Neural Network*)*’* format. Despite the relevance of DL-based plant phenotyping results (such as plant counting, organ detection) to weed recognition research, they are excluded without consideration. Similarly, an extensive review of CNNs for phenotyping by Jiang and Li (2020) used keywords limited to plant phenotyping including ‘plant stress’, ‘plant development’, ‘fruit counting’, ‘flowering counting’, ‘root phenotyping’ and ‘postharvest quality’ to find literature. The approach excluded highly relevant literature (Di Cicco *et al.* 2017) to one of their central conclusions around 3D plant analysis. This is not to criticise the authors; however, it highlights how the accepted norms of each field are hindering development.

The purpose of this mini-review is to highlight overlap in the fields of plant phenotyping and weed recognition in the use of DL for image-based plant analysis. We propose that establishing interdisciplinary ties through open-source work, based on the precedence set by the machine learning community, could overcome current bottlenecks for mutually beneficial results. By evaluating how each field applies DL, we investigate how these typically distinct fields are incorporating similar, if not the same, technologies to address common problems. Given the accessibility and shared use of RGB imaging devices for phenotyping and weed recognition, the focus of the review is on proximal sensing using these devices. That is not to say a similar approach could not also be adopted by multi- and hyperspectral sensors but just that they are beyond the scope of this review. Open-source software and hardware is opening the door for more cross pollination and interdisciplinary collaboration than has been possible in the past. Yet, collaborative outcomes are not inevitable. Open-source software development and research in basic and applied plant sciences must be established as the norm rather than the exception for it to flourish.

## Open-Source Technology

At its core, open-source technology relies upon the availability of source code and data for user-driven development and the free exchange of tools and ideas. The collaborative nature of open-source approaches avoids ‘reinventing the wheel’, as each modification builds on the works of previous developers (Gacek and Arief, 2004). It is hard to ignore the impact of open-source development and technology on the discipline of DL. There are a myriad of open-source Python libraries such as Tensorflow (Abadi *et al.*, 2016) and Pytorch (Paszke *et al.*, 2019) used to build, train and deploy models that operate at a high level of abstraction, so users do not need to rewrite the tedious complexities (or ‘boilerplate code’) of machine learning mathematics. High performing, popular algorithm architectures, e.g. YOLO (You Only Look Once) (Redmon *et al.*, 2016) and SSD (Single Shot Multibox Detector) (Liu *et al.*, 2016), are widely available, have been iterated on many times and are reducing the barrier to entry for high performing CNNs. In the case of the ‘fifth version’ of YOLO, YOLOv5 (https://github.com/ultralytics/yolov5), the state-of-the-art method has over 35 000 ‘stars’ on GitHub, an indicator of its impact. Interestingly and somewhat controversially, the creators of the repository are yet to author a scientific paper on development of the architecture, though a search of Scopus with the term ‘YOLOv5 AND (plant OR weed)’ returns 88 results since its launch in 2021. Ultralytics has since released an eighth version of YOLO (https://github.com/ultralytics/ultralytics) with supposedly improved architectures and ease of use. The popularity is likely the result of ease of use and the high performance achievable on niche datasets (Dang *et al.* 2023). These architectures are typically supplied with pretrained models on large open-source labelled image datasets, such as the COCO (Common Objects in Context) dataset containing over 330,000 images and over 1.5 million object instances. ImageNet pre-training is also widely used. Pre-training for transfer learning allows users to begin more specialised plant/weed related training with an already established model that does not need to learn basic features again, even if retrained on different plants (Bosilj *et al.*, 2020).

The difficulty with bespoke, closed-source systems is new features beyond the original design that require investment are less likely to occur if there is not a sufficient business case; an understandable and logical perspective from a profit-seeking business. A standing feature in agriculture is variability at all levels, thus designing and developing new systems that meet variable use cases (e.g. horticultural, row-crop, plant science) in variable environmental conditions (e.g. weather, soil/plant background, growth stages) is not likely to be reliably served by individual businesses. Moreover, many large companies have transitioned from closed- to open-source approaches to capitalise on the benefits of having diverse communities with many different people working on a project (Kochhar *et al.*, 2019). While there is limited research on generalisability of DL models from one crop and environment to the next, research has found that weed recognition models trained in a broadleaf crop do not translate into cereals (Sapkota *et al.*, 2022). Similar cross-applicability issues have been found with variable environmental conditions such as lighting, soil type and weather (Quan *et al.*, 2019). One advantage of open-source methods is that the community can identify these niche use cases and build from the existing base for their own needs, such as the case for the YOLO series of object detectors (Gehan *et al.*, 2017). This matches well with the complexity of agricultural and plant science disciplines, where it is unlikely that a single system will fit all manners of plant science needs.

The counter to open-source approaches and what often makes researchers and industry reluctant to adopt the approach is the barrier of intellectual property agreements at research institutions and lack of adequate recognition or compensation for contributions (Sonnenburg *et al.*, 2007). For data sharing, the assumption that sharing data results in loss of competitive advantage or losing the research edge on a particular topic conflates the concept of data with information. Data is but the beginning of a process that involves analysis, interpretation, execution, and reflection. The information and science resulting from data is the valuable output upon which future research can be built or decisions made. Beyond data, the ‘development-in-the-open’ of software can be an opportunity to stake a claim in a field during the development process, enabling efficient iteration, adding in layers of redundancy and preventing unnecessary duplication (Prlić and Procter, 2012). For example, the YOLO series of object detection algorithms is now up to ‘v8’, though many other variations exist, all built largely off the initial principle of the original YOLO by Redmon *et al.* (2016). Perhaps in contrast with many research projects that stagnate when a lead researcher leaves, interest in the architecture has flourished given the open-source approach, despite Redmon publicly stating he would not continue working on computer vision research (Synced, 2020) and as a result not continue to develop YOLO. The level of redundancy that this provides is surely enticing for funding bodies that are concerned with ‘wasting’ research funds in projects that reach dead ends.

## Similarities between Phenotyping and Weed Recognition

One defining feature of the ‘deep learning decade’ (2012–2022), has been the availability of many open-source algorithms and public datasets of common objects on which to develop, test and compare. The presence of DL as a backbone in new methods of image-based plant analysis is what drives much of the similarity in needs between phenotyping and weed recognition. For example, counting wheat head prevalence in a crop requires image data collection, wheat head annotation, followed by training and evaluation in Tensorflow (Khaki *et al.*, 2022b). Even though the specifics differ, the general pipeline is related to that of weed detection research (Hasan *et al.*, 2021). Examples of this cross-over are presented in Table 2, where despite semantic differences, the research fundamentals between plant science and weed recognition are common. Khaki *et al.* (2022a) recognise this opportunity for the generalizability of a stand count approach in maize to contribute to weed detection, which could be combined with works such as Picon *et al.* (2022) for maize extraction within weedy environments. Recognising the potential for research overlap, Weyler *et al.* (2021) present a crop-weed detection system, which incorporates leaf counting for growth stage estimation and in-field phenotyping. Given its cross-disciplinary appeal, citations of the article come from both weed recognition and plant science domains; however, the source code is not provided, limiting the ability for more rapid iteration. A pattern of undetected overlap is evident in other approaches such as plant part detection (Mirnezami *et al.*, 2021; Lac *et al.*, 2022), which is often required in the use of highly precise weed control tools such as lasers (Rakhmatulin *et al.*, 2021). Intuitively, the opportunity for phenotyping-weed collaboration is clear, given that a large quantity of research repurposes open-source algorithm architectures (e.g. the YOLO series) built on datasets for common objects. Yet, as observed through what often appears to be the arbitrary development of research silos, the reality seems to be that there is limited direct acknowledgement and practical open-source collaboration occurring.

**Table 2.** Comparison of approaches used within applied precision agriculture/weed control and phenotyping for plant breeding. Algorithm output refers to the primary, intended purpose of the trained algorithm. In weed recognition, this is often simply weed and crop discrimination with varying levels of granularity. Plant phenotyping may require plant organ or part detection, though advanced, novel weed control methods (e.g. lasers) may also require this information.

Discipline	Algorithm output	Plant species	Model	Reference
*Plant detection*				
Phenotyping	Plant stand count *Number of plants)*	Maize *Zea mays*)	DeepStand VGG-16)	Khaki *et al.* (2022a)
Precision weed control	Crop/weed detection	Bok choy *Brassica rapa* subsp*. chinensis*), unspecified ‘weeds’	YOLO-v3, CenterNet, Faster R-CNN	Jin *et al.* (2022)
Precision weed control	Crop detection	Maize	Faster R-CNN	Quan *et al.* (2019)
*Plant part detection*				
Phenotyping	Leaf counting	*Arabidopsis thaliana*	YOLO-v3-tiny	Buzzy *et al.* (2020)
Phenotyping	Leaf counting	*A. thaliana,* tobacco *Nicotiana tabacum*), komatsuna *Brassica rapa* var*. perviridis*)	ResNet50 + regression layer	Giuffrida *et al.* (2018)
Phenotyping	Plant organ segmentation	Unspecified rosebush species	RoseSegNet	Turgut *et al.* (2022)
Phenotyping	Plant node detection	Cucumber *Cucumis sativus*)	YOLO-v3	Boogaard *et al.* (2020)
Phenotyping	Wheat head detection	Wheat *Triticum aestivum*)	WheatNet MobileNetV2)	Khaki *et al.* (2022b)
Precision weed control	Crop-weed detection, leaf counting for growth stage estimation	Sugar beet *Beta vulgaris*), unspecified broadleaf weeds	Custom object detection based on CenterNet)	Weyler *et al.* (2021)
Precision weed control	Stem and crop detection	Sugar beet, ‘dicot’ and ‘monocot’ weeds	Custom CNN	Lottes *et al.* (2020)
Precision weed control	Crop stem detection	Maize, unspecified ‘bean’ species	YOLO-v4	Lac *et al.* (2022)
*Phenology*				
Precision weed control	Growth stage estimation	18 weed species	Inception-v3	Teimouri *et al.* (2018)
Precision agriculture	Growth stage estimation	Mango *Mangifera indica*)	YOLO-v3, YOLO-v4	Roy and Bhaduri (2022)
Phenotyping	Temporal phenotyping	*A. thaliana*	CNN-LSTM	Taghavi Namin *et al.* (2018)

## From Data to Decision

**Figure 1.**
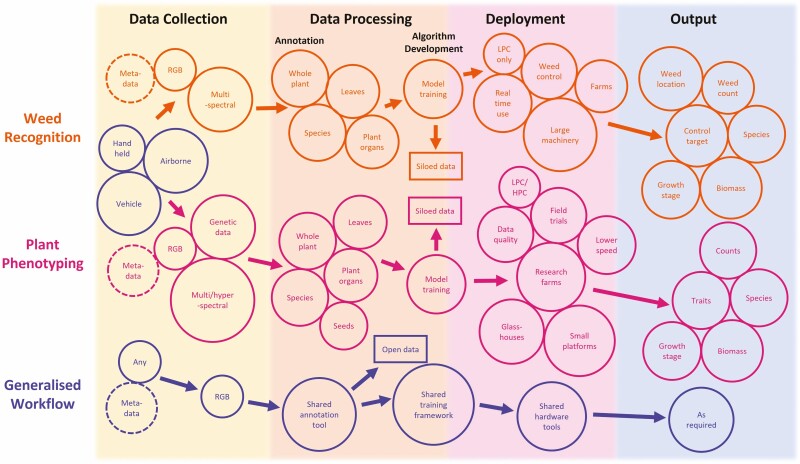
A comparison of deep learning pipelines for weed recognition and plant phenotyping across data collection, data processing (annotation), algorithm development and deployment and algorithm output phases. Many aspects are shared or could benefit from sharing annotation, metadata, dataset, training or deployment infrastructure. Less overlap is apparent in data modalities, the intent of the process and the desired output. RGB refers to red, green and blue digital images collected from cameras. Low-performance computing (LPC) implies use of embedded devices (e.g. Raspberry Pi). High-performance computing (HPC) means data processing is conducted on high-performance equipment where quality/quantity is required. Individual colours denote specific workflows or deep learning pipeline stages. Circles represent individual components of the pipeline clustered around stages, metadata is dashed as an addendum to datasets and boxes represent the output annotated image datasets.

The theme of collaboration, shared uses and mutual benefit is an underlying feature of open-source development. The first step in determining opportunities for collaboration for image-based plant recognition is establishing the entire system, from image data collection through to meaningful output, and defining the areas of overlap that sit within. The steps can be broadly divided into (1) data collection, (2) data processing, (3) model training and deployment, and (4) analysis of algorithm output (Figure 1). In designing a pipeline for a project, the typical approach is to start with the required output, before engineering the rest of the pipeline to meet those requirements. For example, the reality of deploying a weed recognition algorithm for real-time use on field equipment, means resource constrained computing is standard (Chechliński *et al.* 2019). This limitation in turn defines model selection and hence dataset annotation requirements. In the following sections, research and open-source tools relevant to each stage of the process are evaluated.

### Data collection

Image data collection in both weed recognition and phenotyping has been attempted with platforms including tractors (Walter *et al.*, 2019), all-terrain vehicles (ATV) (Laursen *et al.*, 2017; Olsen *et al.*, 2019), push-along carts (Crain *et al.*, 2016), robots (Underwood *et al.*, 2017; Pérez-Ruiz *et al.*, 2020), gantry systems (Le *et al.*, 2019) and handheld cameras or phones (Ngugi *et al.*, 2020). The Bonirob agricultural robotic system was initially proposed for in-field plant phenotyping (Ruckelshausen *et al.*, 2009), the name based on the German word ‘bonitur’ meaning ‘rating’; however, the project transitioned to collection/use of weed image datasets (Haug and Ostermann, 2015). Laursen *et al.* (2017) developed an ATV mounted camera system (RoboWeedSupport) that collects high-quality image-data at up to 50 km h^−1^ with sub-millimetre resolution. Portions of the data collected have been published online (https://weed-ai.sydney.edu.au/datasets/aa0cb351-9b5a-400f-bb2e-ed02b2da3699). Ground-based systems for plant phenotyping have been reviewed previously (Xu and Li, 2022), with the authors concluding that dedicated systems were often prohibitively expensive to obtain for most plant breeding programs. Further, these systems are identified as too slow for large-scale breeding operations. Yet, it is becoming more common place for commercial targeted weed control systems to have self-propelled sprayers fitted with cameras at consistent spacings up to 55 m wide (Martin, 2021). While these cameras are used to detect weeds, there is also an opportunity for the concurrent rapid collection of large, albeit private quantities of image data with associated weed species and location information. The spatial/temporal scale and quantity of information collected on weed prevalence is not something that has previously been possible, an exciting prospect for understanding weed ecology.

Open-source platforms and hardware for data collection exist, though often have tangentially related purposes that require alteration for the intended use. The Acorn robot from Twisted Fields (https://github.com/Twisted-Fields) is a recently open-sourced robotic platform that may prove useful in both weed control and phenotyping disciplines, however, it has not yet been widely used. The open-source GPS system, AgOpenGPS (https://github.com/farmerbriantee/AgOpenGPS), is providing an additional development pathway to autonomous field robots by removing the requirement for expensive self-driving and closed-source GPS systems. Location data could be integrated with image data collection using such a system; however, it is unclear if this has been used in research platforms to date, given the largely farmer-focussed user base of AgOpenGPS. Robotic gantry systems such as Farmbot (https://github.com/FarmBot) could serve as low cost and customisable systems for data collection in fixed semi-field settings. For data collection, the recently released OpenWeedLocator (OWL) project (Coleman *et al.*, 2022) provides a method for image collection and integration with colour-based detection algorithms (https://github.com/geezacoleman/OpenWeedLocator). While developed with weed detection in mind, the tool could be adapted for image data collection. Integration with low-cost GPS systems for location data is currently in progress. Whilst there are some open-source data collection hardware available, adaptation for phenotyping or weed recognition may pose a challenge.

Whilst image data collection is critical, it should be supported and contextualised with metadata for reproducibility and potential needs of future users. A major theme from reviews identified in Table 1 was developing standards and sharing standardised image datasets with associated metadata. These standards must be acknowledged and adhered to at the collection stage, so important attributes are not missed.

### Data processing and availability

After image data are collected, images must be annotated with relevant attributes for the training of DL algorithms to recognise the same patterns in unseen data when deployed. The form of detection output, and therefore the annotation approach, should be determined by the end use of the algorithm. Weed control tools that require highly precise targeting and positioning such as lasers and electrical weeding must have information on specific plant organs to target (Rakhmatulin *et al.*, 2021). This is similarly true for a DL algorithm to reliably measure important plant phenotypic traits, such as wheat head counts, flower presence or disease severity ratings. For state-of-the-art SSWC uses and many plant phenotyping tasks this necessitates the annotation of not just individual plants in an image but also plant organs, growth stages and plant species. The laborious image annotation process and access to adequate datasets required for robust algorithm training is widely considered to be the largest bottleneck to adoption of DL techniques not only in the plant and agricultural sciences but in science more generally (Table 1). Building shareable, reproducible and open-source datasets requires development and adoption of metadata and image data standards.

**Figure 2.**
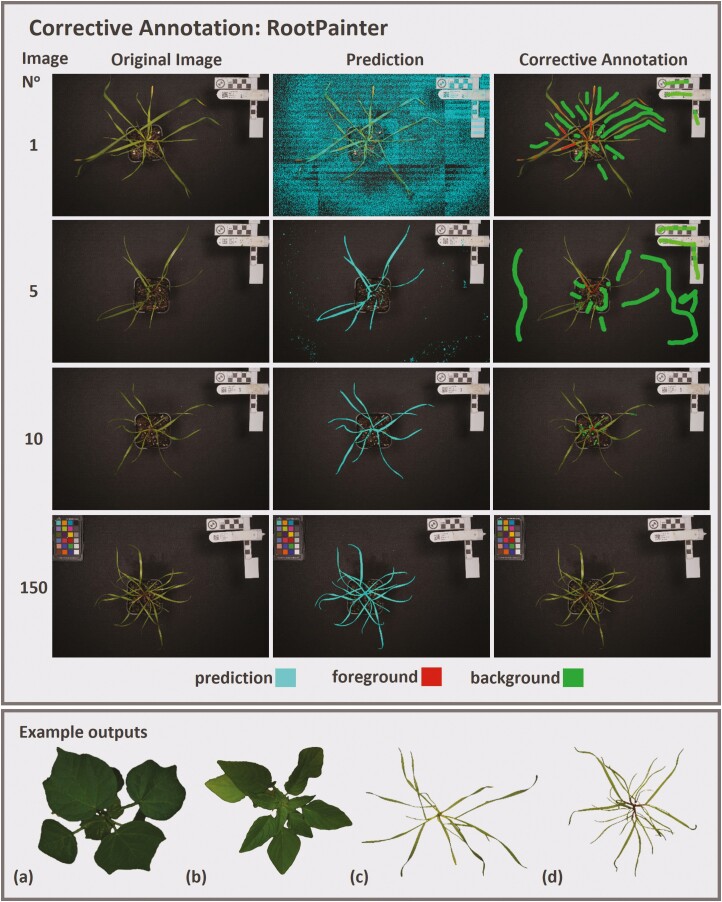
Example of a corrective annotation approach provided with the RootPainter (Smith *et al.*, 2022) open-source software. As more annotation data are provided (image 1, 5, 10 and 150 shown) the performance increases without requiring the manual annotation of an entire image. The cyan prediction in can be corrected in with only minor changes and relatively few images for training, without the need to annotate areas of high confidence. Example outputs of cotton (*Gossypium hirsutum*), Palmer amaranth (*Amaranthus palmeri*), wheat (*Triticum aestivum*) and rigid ryegrass (*Lolium rigidum*) are provided in the bottom box, which contrast with the original intent of RootPainter for root phenotyping.

Many annotation tools exist, as outlined by Lu and Young (2020), including many open-source platforms already in use for phenotyping research (Boogaard *et al.*, 2020; Buzzy *et al.*, 2020; Lu *et al.*, 2022). This combined need and existing but siloed use of tools for annotating image data highlights potential benefits for many plant science fields if generalised, open-source, and standardised approaches are adopted. For example, RootPainter (Smith *et al.*, 2022) was initially developed as an open-source tool for rhizotron-based root architecture research; however, the software has proven equally useful in segmenting above-ground leaf and stem material of rigid ryegrass (*Lolium rigidum*), Palmer amaranth (*Amaranthus palmeri*), cotton (*Gossypium hirsutum*), and wheat (*Triticum aestivum*) (Figure 2). The method uses a U-net architecture trained with corrective annotation, allowing a reduced effort approach. Our experience with the method has been positive, requiring the annotation of approximately 150 images (about three to four hours of work) per species to develop the outputs in Figure 2. Our annotation quality needs were particularly stringent and moderate results would have been achieved with fewer images. It provides options for use on the free (albeit with restrictive usage limits) Google Colaboratory platform or on local GPUs, and the easy-to-use interface improves accessibility for non-computer science research groups. While just one example of an open-source approach advancing science beyond its intended field, the benefits of such an approach for other tools that improve annotation for plant and agricultural science tasks is clear.

Open access to annotated image datasets is critical for confirming performance, developing new models and benchmarking against previous work. The Imagenet large scale visual recognition challenge (ILSVRC) was developed as a competitive approach to accelerating algorithm development (Russakovsky *et al.*, 2015). It played a pivotal role in demonstrating that CNNs performed better on larger, complex datasets than previous non-deep methods (Krizhevsky *et al.*, 2012), paving the way for the DL decade and subsequent benefits in plant science. Plant-based benchmarking datasets are thus critical for both fields of research. One example has been the DeepWeeds dataset, which has been the basis for new architectures (dos Santos Ferreira *et al.*, 2019; Hu *et al.*, 2020). Shared development of a highly detailed dataset for growing point detection in weeds, would be equally beneficial for phenotyping. Large projects dedicated to this pursuit, must be cognisant of the wider community that would benefit from such infrastructure, capturing research and interest from more people, likely accelerating development. One example of an open-source platform is Weed-AI (https://weed-ai.sydney.edu.au/about). Weed-AI is an open-source image sharing platform for weed recognition, built around standardised metadata (in an ‘agcontext’ file) and annotation formats. The ‘agcontext’ is appended to a COCO (common objects in context) annotation file in a singular ‘weedCOCO’ annotation file. It integrates with tools that support the widely used COCO format for annotations, whilst incorporating a standardised metadata reporting system. The platform also integrates the open-source computer vision annotation tool (CVAT) for public annotation of unannotated images. Whilst built for weed recognition data, it may provide useful opportunities and basis for plant phenotyping.

### Model training and deployment

A drawback of model training is it can be art as much as science. With many different ‘hyperparameters’ to adjust, the somewhat opaque understanding of underlying algorithm attention and dataset variability, large changes in performance can occur with small differences in code. Simply changing framework and keeping architecture consistent, Hussain *et al.* (2021) found a 1% improvement in performance with Pytorch over Tensorflow. Without sufficient reporting and oversight of the approach (e.g. data, code, and configuration), reproducibility is diminished. A standardized platform for this critical step may help, where research often appears to be conducted with bespoke implementations of each algorithm (e.g.Dang *et al.*, 2023; Olsen *et al.*, 2019).

The AgML project (https://github.com/Project-AgML/AgML) is an open-source platform designed to assist the training of machine learning models in agriculture, providing a standardised format for managing data and models (Joshi *et al.*, 2022). A common platform, specific to agriculture, with a consistent data management, training and evaluation pipeline would greatly benefit the efficiency of weed recognition and plant phenotyping DL research and development. The seventh benefit of open-source approaches outlined by Sonnenburg *et al.* (2007) is the improved ability to standardise approaches. Having a shared platform on which to test and train expedites this standardisation. Other platforms such as Roboflow (https://roboflow.com/), Huggingface (https://huggingface.co/) and Weights and Biases (https://wandb.ai/site) are providing non-agricultural specific methods of managing model training. The logging of training process could also manage reproducibility issues with version control of open-source software and datasets. Whilst platforms like GitHub and Weed-AI provide version history, publications should ensure that access dates and version identifiers of datasets and code bases are cited accurately.

The differences between disciplines emerge at the deployment stage of DL models. Where phenotyping may require high quality outputs integrated with trait information for further analysis, the in-field deployment of conventional SSWC systems (e.g. spot sprayers) can accept much lower precision. Yet, more advanced non-chemical methods (e.g. lasers) or more targeted weed control of the future (e.g. growth stage or plant biomass-based control) may have a comparable level of required information output. A trend towards phenotyping individual weeds is becoming apparent, as decisions are made in real-time about the risk posed to crop yield from herbicide resistance status, competitive ability or beneficial plant (Gerhards *et al.*, 2022). Nevertheless, emphasis on error type may differ. For a farmer, a missed weed (false negative) is generally more consequential than overusing the input. In contrast, understanding the effect of detection sensitivity on data quality is critical in plant phenotyping where data reliability and error sources may impact trait selection.

Open-source, post DL processing tools, such as PlantCV (Gehan *et al.*, 2017), can provide morphological information on segmented plants, such as leaf angle, leaf counts and architecture. Besides analysis, PlantCV provides some initial image processing tools to extract the plant from the background, however, does not use DL. The platform is valuable for rapidly phenotyping large numbers of plants imaged under consistent conditions, however, is less useful for entirely field-based work. So, while it is valuable for understanding the morphology of weeds for weed science purposes, there is less benefit for applied, real-time SSWC. Tools in this part of the process are less likely to be candidates for cross-disciplinary collaboration; however, open-source approaches would leave the door open for works that adapt it to other, unexpected purposes.

## Discussion and Conclusion

Through this mini-review, we have highlighted substantial areas of overlap DL-based plant analysis between weed recognition and plant phenotyping. Domain-specific reviews unintentionally exclude highly relevant research due to varying semantics of weed recognition and plant phenotyping, despite both essentially focused on image-based plant analysis. The level of seemingly invisible overlap only reinforces the need for more open-source collaboration. One of the main purposes of open-source development is its ‘bigger-than-the-project’ influence and reach. Funding bodies should consider the wider potential of open-source approaches when considering projects. While the temptation is to keep intellectual property closed, if the outputs are not used and remain behind closed doors, then any benefit is effectively nullified. Open-sourcing outputs from publicly funded sources ensures that the public can access project outputs irrespective of commercial value and develop or benefit in ways that were not envisaged at the outset. The pathway to successful adoption of open-source approaches in plant phenotyping and weed recognition need not be a reinvention of the wheel. Through the ideas presented in Box 1, the wider machine learning community has successfully demonstrated that open-source development and lucrative commercialisation are mutually agreeable. There are three key messages from this field that resonate with the gaps identified in the phenotyping and weed recognition domains, namely: (1) development of shared data standards and platforms; (2) access to large datasets for benchmarking and development; and (3) recognition of contributions to open-source development.

Open-source outputs are undervalued in official academic recordings of research outputs and engagement, where scholarly metrics are calculated from publication and citation counts. It seems logical to incorporate other forms of engagement such as ‘stars’ or ‘forks’ on GitHub and open-access dataset citations on platforms such as Zenodo. The purpose of a citation is recognising the contribution of previous work to the current research; enabling software should receive this recognition, and contributions that support science should have the opportunity to be acknowledged. The conversation around incentivising and recognising open-source software, hardware and dataset contributions in academia should be part of any plant science conference. Particularly given that most results shared at such events would have likely been analysed, illustrated, and perhaps collected with open-source software and packages. The question has been raised previously (Danilevicz *et al.*, 2021); however, a simple solution is not clear.

Given the similarities, this review has focused on the fields of weed recognition and plant phenotyping only; however, due to the nature of DL methods, it would be logical to incorporate approaches from other precision management tools such insect and disease detection. Similar metadata, standardisation and data requirements are likely equally important for research in these fields. After all, an image is just a matrix of numbers, the annotation and context of use is what drives meaning and specificity. A novel area of research would be developing combined approaches for plant analysis that incorporates all pest management and phenotyping information for decision making. A combined field-scale dataset would be needed to train algorithms on weeds, insects, diseases and plant phenotype concurrently. Equally, from a phenotyping perspective, a wholistic picture of biotic stresses on plants during the season would help explain trial variability and improve research outcomes. These multi-discipline datasets will likely be the way of the future for image-based plant science research; however, require interdisciplinary collaboration.

The accessibility of DL tools has enabled technology to advance and permeate into areas such as plant science that may otherwise lack the intrigue of computer vision researchers. While using ideas from different fields is certainly not new, and in fact a fundamental strength of science, open-source technology is reimagining how this can and should occur. Given the awareness of these benefits and use cases for shared tools, there is a need to consider alternative research uses at the outset of software development. Opening development to a community through platforms such as GitHub accelerates how this occurs, giving users from different fields the capacity to contribute to the direction and features of software. Whilst there are often strong distinctions made between applied and basic sciences, the use of open-source tools is bringing these fields closer together than has previously been possible. The concept of ‘one person’s weed is another’s crop’ (Harlan, 1982) has interesting parallels here. Phenotyping is often associated with crops, not weeds but the principles used in both are highly related. Standing on the shoulders of giants is likely easier if all giants involved in the process are aware of each other and walking in similar directions.

## Data Availability

Data sharing not applicable to this article as no datasets were generated or analysed during the current study.
